# Individualised placement support as an employment intervention for individuals with chronic pain: a qualitative exploration of stakeholder views

**DOI:** 10.3399/bjgpopen20X101036

**Published:** 2020-07-01

**Authors:** Michelle M Holmes, Sabina C Stanescu, Catherine Linaker, Catherine Price, Nick Maguire, Simon Fraser, Karen Walker-Bone

**Affiliations:** 1 Psychology, University of Southampton, Southampton, UK; 2 Medical Research Council Lifecourse Epidemiology Unit, Southampton General Hospital, University of Southampton, Southampton, UK; 3 Arthritis Research UK / MRC Centre for Musculoskeletal Health and Work, Southampton General Hospital, University of Southampton, Southampton, UK; 4 School of Primary Care and Population Sciences, Southampton General Hospital, University of Southampton, Southampton, UK; 5 Solent NHS Trust, Southampton, UK

**Keywords:** return to work, rehabilitation, occupational health services, employment, chronic pain, musculoskeletal pain, primary health care

## Abstract

**Background:**

Individualised Placement and Support (IPS) is a tailored, client-centred employment intervention for people with chronic health conditions. It involves the integration of vocational advisers within health teams to optimise return-to-work strategies. The intervention aims to get clients into employment by complementing traditional job searching skills with placements, and one-to-one mentoring alongside a work-focused health intervention.

**Aim:**

To explore the concept of IPS for individuals with chronic pain.

**Design & setting:**

A multi-method qualitative study was designed to explore stakeholder views of IPS for individuals with chronic pain in southern England.

**Method:**

Fourteen semi-structured interviews and three focus groups were conducted with current recipients of IPS (clients), employment support workers (ESWs), and healthcare professionals (HCPs). All data were audio-recorded, transcribed, and analysed using thematic analysis.

**Results:**

In total, 11 HCPs, five ESWs, and nine clients participated in the study. The analysis identified four themes. The situations of chronic pain patients were discussed, including their complex needs, multifaceted relationship with work, support from HCPs, and existing programmes that were failing to meet their needs. The intervention input was highlighted, including the recruitment procedures and role of ESWs. Programme activities and outcomes were also identified.

**Conclusion:**

This study identified the complex needs and relationship with work of individuals with chronic pain. It showed that ESWs need to understand the unpredictability of symptoms for individuals with chronic pain and that clients may need additional support before a placement. The findings highlighted several activities for future IPS interventions and potential outcomes for future evaluation.

## How this fits in

Chronic pain significantly impacts individuals’ ability to work, but unemployment leads to additional health problems. IPS is a suggested approach to aid rehabilitation of unemployed individuals with chronic pain into work. This study explored the complex needs of chronic pain patients and their multifaceted relationship with work. The results suggest an integration of clinical practice and employment support, aiming to facilitate the rehabilitation of people with chronic pain into work.

## Introduction

Chronic pain, defined as 'pain that persists past normal healing time' (usually taken as 3 months), can significantly impact an individual’s quality of life.^1,2^ Chronic pain is estimated to affect approximately 20% of people worldwide,^1^ with one in five adults in Europe thought to have persistent pain.^2^ In the UK, chronic pain affects an estimated 43% of the population.^3^ Approximately 20–27% of people of working age are unable to participate in their usual activities, including work, due to chronic pain.^4^ Prolonged unemployment causes additional health problems^5^, including poorer mental health,^6^ poorer life expectancy,^7^ and frequent consultations with healthcare professionals, reporting higher levels of pain.^8^ People with chronic pain report that they often wish to work and have called for more support to enable them to work.

One well-used model to rehabilitate individuals into employment is the ‘train-and-place model’. Within the train-and-place model, clients participate in vocational assessment, work training, and job placement.^9^ However, there is little evidence showing that this model is effective for employment interventions for chronic pain patients. An alternative model is the ‘place-and-train model’, in which clients are placed into employment, followed by training and support while in employment.^10^ The most researched approach using the place-and-train model is Individualised Placement Support (IPS). The IPS approach focuses on helping clients obtain employment, based on their preferences and clinical needs, with continued support from a trained vocational adviser.^11^ There are several core principles for IPS: attention on client preferences and needs, focus on competitive employment, rapid job search, integration of medical rehabilitation, continuous assessment, and individualised support.^11^

IPS has been most carefully evaluated in people with severe mental health conditions and is now evidence-based best practice to help people with severe mental illness gain employment.^12^ Individuals with chronic pain also suffer from disability and social isolation, comparable to that experienced by individuals with severe mental illness,^13^ and have high levels of psychological comorbidity.^14,15^ Given the success for individuals with mental health conditions, IPS may also be a successful strategy for patients with chronic pain.^16^ The principles of IPS may be beneficial to individuals with chronic pain, assisting them to gain employment through identifying their needs, integration with pain services, and individualised support. The overall objective of this study was to explore needs and benefits in relation to IPS for patients with chronic pain, as well as relevant stakeholder views of the intervention.

## Method

This research adopted a pragmatic philosophy allowing for an outcome-orientated approach, using a multi-method qualitative study to explore individuals’ views and experiences of IPS.^17^ Interviews and focus groups were conducted with stakeholders who are involved with the IPS programme in southern England: clients, employment support workers (ESWs), and healthcare professionals (HCPs). Two female PhD students with prior experience in qualitative data collection (MH, SS), who had no prior relationship with the participants, developed the data collection tools, and completed data collection and analysis. All participants gave informed consent to take part in the interviews and focus groups.

### Recruitment

Participants were recruited within the south of England, using convenience sampling. Clients with physical health conditions, currently attending an IPS programme, were invited to take part in an in-depth interview. ESWs were also invited to take part in an interview. Primary HCPs were contacted through the local Clinical Research Network, with two primary care practices agreeing to participate. HCPs from these primary care practices were then invited to take part in focus groups to explore their views of IPS and the feasibility of doing a trial of IPS that would recruit patients with chronic pain through primary care. Recruitment continued alongside data collection and analysis until data saturation was reached (no new themes were generated from the data), which was assessed by two authors.^18^

### Data collection

Semi-structured interviews and focus groups were conducted between March and August 2017, following a semi-structured topic guide (see Table 1 for topics). Open-ended questions and prompts were developed to help the interviewer achieve the research aim, but this was flexible to allow for changes to be made throughout the process and encourage discussion.

**Table 1.** Topics and example questions used in interviews

**Clients**	**Employment support workers**	**Healthcare professionals**
Views around health and employment (*for example, what are your thoughts about returning to work?*) IPS recruitment processes (*for example, how did you get to be involved in the programme?*) Programme activities (*for example, what activities have you been involved with?*) Benefits of the programme (*for example, what do you think you have got out of the programme?*)	Role of employment support workers (*for example, can you tell me about your role in the programme?*) Programme goals, activities, and outcomes (*for example, can you walk me, step-by-step, through your involvement with a client?*) IPS recruitment processes (*for example, what do you think about the recruitment process?*) Integration with other programmes and other pain services (*for example, what support do you think you need to work with existing services?*)	Benefits of IPS (*for example, what do you think are the benefits of IPS for those who are unemployed due to chronic pain?*) Integration of IPS and other pain services (*for example, how could IPS work with primary care and existing services?*) IPS recruitment processes (*for example, what are your thoughts on identifying and recruiting patients with chronic pain who are unemployed?*) Evaluation of IPS (*for example, can you think of any other issues to the intervention?*)

IPS = Individualised Placement and Support.

Using qualitative interviews and focus groups allows for exploration of stakeholders’ subjective evaluations of participating in a trial and using IPS.^19,20^ Focus groups were used to facilitate interaction between participants, allowing them to bounce ideas off each other.^21^ Focus groups were helpful for the HCPs, enabling them to discuss what happens in their practice and the different roles they have. One focus group was conducted with clients for their convenience. Interviews were used to allow for participants to express themselves, giving individuals a chance to tell the story of their experiences.^22,23^ All interviews and focus groups were conducted face-to-face at a time suitable for the participants. Interviews and focus groups with clients and advisers were conducted at local IPS sites; focus groups with HCPs were conducted at their place of work. Interviewers explained that they were independent to the IPS programme and no one from the IPS programme was present during data collection. Field notes were made by the interviewers during and immediately after the data collection.

### Data analysis

Interviews and focus groups were audio-recorded and transcribed by two interviewers, before being imported into the computer-assisted qualitative data analysis software NVivo (version 11) for analysis.^24^ The data were analysed using thematic analysis as an iterative process alongside data collection.^25^ Quotes have been selected from the arising themes to best describe the findings, with ID numbers given to participants to ensure anonymity. The study findings are reported according to the standard for reporting qualitative research.^26^

## Results

Two GP practices were contacted; both agreed to take part in focus groups. Twenty-four clients who were engaged with IPS were contacted, of whom 12 showed interest in participating in the research. Clients were not pressured into giving reasons for non-participation, and there is no information on potential differences between them and interviewed participants. A total of three focus groups and 14 interviews were conducted, encompassing 11 HCPs (all women), five ESWs (two men, three women), and nine clients (five men, four women). The mean times for focus groups and interviews were 46 and 29 minutes, respectively.

**Figure 1.**
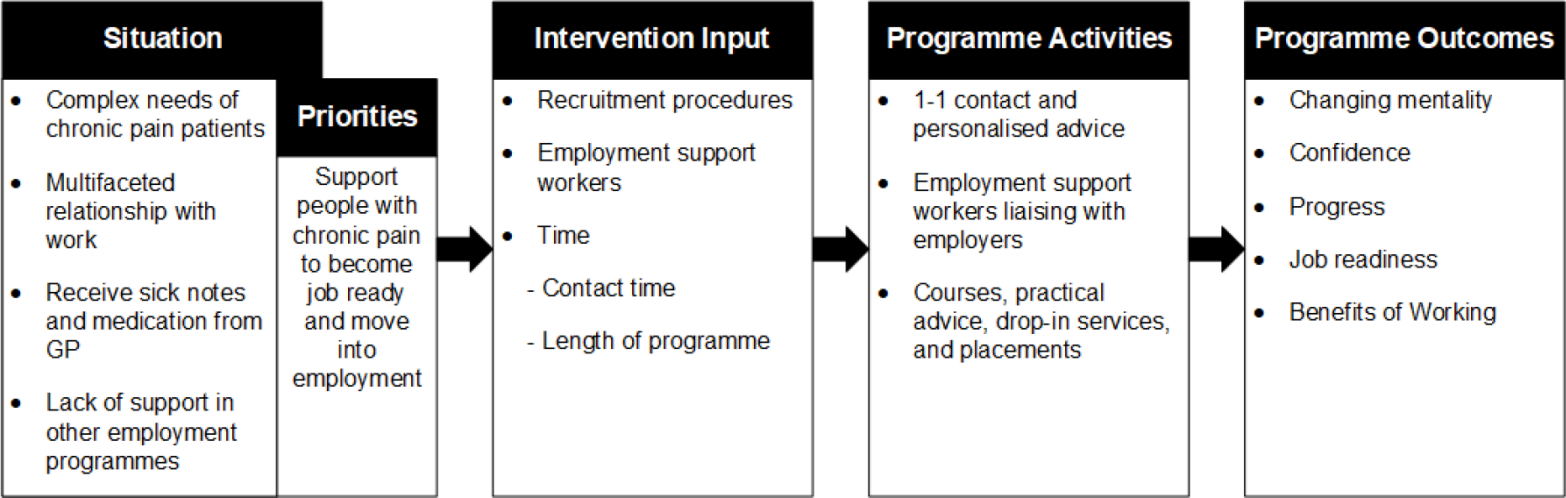
Logic model of Individualised Placement and Support

The interviews aimed to explore the experience of IPS for current clients and ESWs, as well as to explore views surrounding IPS for individuals with chronic pain, from clients, ESWs, and HCPs. The overall analysis identified four themes: situation of chronic pain patients, intervention input, programme activities, and programme outcomes. The following sections describe each theme, noting stakeholder views, with example quotations from participants. A logic model was created depicting the four themes and subthemes (see Figure 1).

### Situation of chronic pain patients

#### Complex needs

ESWs identified that patients’ flare ups of pain were difficult to manage and prevented them from remaining in work, despite the patients' desire to work. ESWs and HCPs discussed the need for planning and managing patients’ pain, including pacing, stress management, and emotional support. It was noted that people with chronic pain would have different work requirements:

*'we just have to make sure we are pacing everything right for her. That she was going into a flexible environment, so if she needed to sit down that she would be able to*.' (ESW1)

Clients spoke about the ‘boom and bust’ cycle of overexerting themselves on days they felt well, completing activities to a perceived level of normal:

*'And you can push through maybe a day and you’d probably be ok the next day if you don’t do too much, but by the third day you’re in bed*.' (C5)

HCPs had concerns that patients held deep-seated beliefs that may influence their view of work. They discussed the *'vicious circle*' of chronic pain, acknowledging that it could be incapacitating for some individuals. There were concerns that some patients do not wish to change the management of their pain or coping strategies, for fear their pain or functioning may worsen:

*'There are also people who become institutionalised in their own self-management*.' (HCP2)

It was also felt that chronic pain could be rewarding for some individuals, both financially and for their lifestyle, and this could lead to people not wanting to change or improve their health:

*'Our culture allows people to revel in the sick role a lot.'* (HCP1)

#### Relationship with work

All three stakeholder groups alluded to the importance of the relationship between the client and their motivation to work. HCPs identified three groups of patients: those who are keen to return to work; those who could return to work if their work was modified due to their condition; and, those who do not want to return to work. Clients also spoke about wanting to return to work:

*'I have been banging my head against the wall for a while, trying to get myself back into employmen*t.' (C4)

All participants identified concerns about clients returning to work, and not knowing what employment they could now enter, due to their conditions:

*'I'm not scared of work. I've always worked. I've worked ever since I've left school. It's only because I got the injury, so I've got to adapt now.'* (C1)

Some clients were still not sure if they wanted to return to work:

*'So, the cost to my personal life, for the benefit of me working, my pain would get worse, my tiredness would get worse*.' (C5)

Additional reasons for lack of work were identified, such as the economic climate and state of industry. Stakeholders discussed the complex issues regarding state benefits:

*'Because I was gonna go into work for them, but I would’ve been financially a lot worse off.*' (C8)

#### Role of health care professionals

HCPs discussed their role in helping patients return to work:

*'We are just medicalising their pain in terms of either prescribing analgesics or whatever and signing them off*.' (HCP4)

Due to time constraints, they felt that they could contribute little into helping patients back to work. They felt their role was mainly around diagnosis, pain management, and providing medical certificates. However, they felt that more could be done for patients in this situation:

*'my feeling is, as a clinician, to give a sick note is ... I really think I would give it as part of a treatment regime.'* (HCP1)

HCPs suggested medical certificates should be part of a package of care, providing support and information in returning to work.

#### Current employment programmes compared to IPS priorities

Clients and ESWs were aware that there were a variety of employment interventions available, but considered many of them inappropriate for individuals with complex issues:

*'The other programme, they understood my problems but they didn’t do anything about it.'* (C4)

IPS was seen as a different approach, helping participants with a health condition identify their limitations, become ‘job-ready’, and support them into employment. The ESWs described the programme as giving clients time, continuous support, and tailoring the programme for them:

*'I love my job. Because I know it works, I can honestly speak to clients, ”yes it does work“, and it does.'* (ESW3)

### Intervention input

#### Identification of unemployed people with chronic pain wishing to return to work

Stakeholders identified a range of ways in which eligible patients could be identified. The existing IPS intervention was recruiting through the job centre, but stakeholders felt that these patients could be identified elsewhere, including GP practices, local chronic pain services, physiotherapy services, rheumatology departments, support groups, community groups, and libraries. HCPs also felt they could personally identify individuals who would benefit from the programme.

Clients had varying expectations of the programme, although some were hopeful about it helping them, others were more sceptical:

*'I'll do whatever they ask of me, but … what's going to come out of it? Or is it just a waste of time?*' (C1)

There was confusion among clients on the voluntary or mandatory status of the programme. Although the programme was explained by the ESWs as being voluntary (IPS is designed for people who want to work), some clients had not felt that they had free choice when referred by the job centre. HCPs recognised the importance of client engagement with the intervention if it was to be successful. Clients emphasised the importance of providing good information about the programme and its aims before attending for the first IPS session:

*'They didn't tell me why or anything, didn't know who I had to see. No one knew why I was here. So it was a bit… there was no explanation why I was sent here or what it's for*.' (C3)

#### Employment support workers

Other clients felt that they had experienced little stability in the programme and not developed a relationship with any one adviser, having met multiple ESWs; this was felt to hinder their progress and undermine their experience of IPS. ESWs acknowledged that, although they tailor the programme to their participants, this was based on the discussion with clients. They recognised that, as non-healthcare professionals, they had only limited knowledge about the impact and management of chronic health conditions:

*'It’s like a group, obviously workers, but like a family, they work well together.'* (C9)

*'You should have advisers that have training around chronic pain and there should be a fully comprehensive directory of signposting people.'* (ESW4)

#### Timing

The existing IPS programme was offered to individuals with chronic ill health after 2 years of unemployment. All participants acknowledged that the sooner someone could enter the programme, the more it was likely to benefit individuals. Long waiting lists were considered a disadvantage of other interventions:

*'Once they've made the first step to talk to us about it. Or they've put their hand up and said ”yes, I do need some help” they then have to wait for months to get that intervention, is quite demoralising for a lot of people*.' (HCP2).

Clients and ESWs also felt the programme could, in some cases, need to involve support for longer than a year, as it could take longer to get someone 'employment ready' and into a job.

### Programme activities

#### One-to-one contact and personalised advice

One-to-one contact was seen as the most important aspect of the programme. ESWs were seen as supportive and friendly in the way they communicated with clients:

*'They’ve got the experience and the attitude about what they need to support you, but they’re good at listening and they’ll listen to your problems and they’ll work out a solution.'* (C4)

Alongside the one-to-one contact, clients appreciated peer support, if they could talk to someone who had already been through the same issue, and guided self-learning. ESWs and clients had to build rapport for the programme to work, and clients had to be committed to the programme:

*it’s critical that we start to have that trust* […] *Once that trust is instilled, the customer will come along on the journey with us.'* (ESW4)

ESWs also identified that they work holistically:

*'I make a thing of the person. Who is being listened to, as is right and proper. That’s the key. Forget ticking and dotting, you look at the person.'* (ESW3)

Clients also discussed the personalised advice and support they were given, feeling that the ESWs were looking out for their best interest, by identifying their potential, their skills, their needs and barriers in to create an individual plan.

#### Liaison with employers

Clients felt that one of the key roles of the ESW was to liaise with employers:

*'She can bridge that gap. So, having an adviser who’s liaising with people who are looking for employees is great, because there is an understanding.*' (C5)

ESWs acknowledged this role, but also identified that more could be done to collaborate with other agencies. They felt that a job was possible for all clients:

*'I’m racking my brains, there is always something somebody can do.'* (ESW3)

They did, however, find difficulties: in general, more employers needed awareness of the potential of people with chronic ill health, and they felt that government could do more to promote work participation among people with health conditions.

#### Courses, practical advice, drop-in services, and placements

Clients described a number of courses they could undertake to improve their skills and employment opportunities. However, there was often a limited range, and courses were not always relevant to individuals on the programme. Clients appreciated the practical advice given to them by ESWs and during drop-in sessions, such as advice on applying for jobs, editing their CV, completing cover letters, practice interviews, and state benefits:

*'I don’t have a time limit* […]*, none of it is rushed, I can take my time, I can concentrate, and that’s the key thing, when you’re on this type of programme.'* (C4)

ESWs discussed supporting clients into a paid placement scheme, which is tailored to each client. Only one client in this study had completed a placement, which he felt was not personalised and felt unsupported within the role:

*'I said I couldn’t do it no more. I didn’t feel safe.'* (C2)

### Programme outcomes

#### Changing mentality

HCPs talked about the necessity of changing patients’ attitudes towards work. The ESWs found through the support of the programme, identifying employment options, tackling social isolation, and improving confidence and self-worth, clients’ mentality towards work often changed:

*'They can see it’s realistic for them to move into work, whereas at one time they might have thought it’s really hard, they won’t achieve it, but they are just realising now it’s quite realistic and they could do it.'* (ESW5)

Clients also spoke about themselves changing throughout their time on the programme:

*'I think the programme itself is fine, it’s just me, I need to take on a complete change. I’m doing my bit now, I got a different attitude.'* (C4)

#### Confidence

HCPs and ESWs discussed the need to build clients’ confidence for them to return to work:

*'They find it difficult to go to these things, and they need more of a one-to-one relationship to build up their confidence to be able to get to that stage*.' (HCP11)

ESWs discussed how some clients have the skills to return to work but, due to their unemployment, have lost confidence in their skills and ability to undertake work. This was seen as a key benefit of the programme.

*'But yeah, I'm now confident so if I do get something I can move forward, even if it's just part time. I feel confident about finding something*.' (C2)

#### Progress

Although the outcome of the programme was to find paid employment, ESWs and clients discussed the progress that clients would make:

*'And, yeah, they all seem to get something out of it. And we do try and make sure they feel like they are achieving.*' (ESW1)

As the clients were still participating in the programme, none of the clients had yet found paid employment, however, clients were keen to discuss their progress:

*'I’ve done so much this year, it’s unbelievable. I can look back and think I’ve had a really good year actually … And there’s still a long way to go, but it’s positive.'* (C9)

#### Job readiness

Clients discussed the skills they had gained from the programme that would make them qualified for employment, such as job searching techniques, practice interviews, improving CVs and cover letters, and qualifications in new employment areas. Clients reported that developing confidence and communication skills was also getting them ready for employment, and helping them understand and overcome the personal challenges they would face:

*'At first I was a bit sceptic, thinking about what sort of office work I could take, I was never any good at admin work, I would say I am intermediate now.'* (C4)

#### Benefits of working

HCPs discussed the benefits of working for individuals with chronic pain:

*'The less they do, the more their pain is. That's the benefit of being more active. That does reduce their pain level*s.' (HCP5)

HCPs and ESWs also discussed the link between chronic pain and mental health, highlighting that many individuals with chronic pain also have symptoms of depression. It was suggested that working can help depression, by improving social interaction, and giving individuals a sense of achievement and accomplishment, *'* *a degree of hopefulness* *'* (HCP8), and improved quality of life.

## Discussion

### Summary

This study aimed to explore IPS as an employment intervention for individuals living with chronic pain. Interviews and focus groups with clients, ESWs and HCPs were conducted to explore their views. It was clear that chronic pain patients had many interacting needs and a multifaceted relationship with work, with varying levels of motivation for returning to work. The existing IPS programme followed IPS principles regarding programme activities, such as support from one-to-one advisers, but additional positive elements were identified. A series of programme outcomes were acknowledged from the programme, including changing mentality, increasing confidence, and improving job readiness.

### Strengths and limitations

This study provides a rich description of IPS for individuals of chronic pain. Data were collected from three stakeholder groups — clients, ESWs, and HCPS — to capture different perspectives. Two authors were responsible for coding the data, creating and revising the code book, and interpreting the data. This reduced the level of subjectivity of the analysis, which increased the trustworthiness of the findings. During recruitment, every care was taken to remind participants that the two interviewers were not part of the IPS programme, however, participants may have viewed the interviewers as part of the programme. A number of clients showed initial interest in participating in an interview, but then either did not respond to contact or failed to attend the agreed time for the interview. This sampling of participants may limit the transferability of the results.

### Comparison with existing literature

Participants identified that individuals with chronic pain had complex needs. Chronic pain patients have to learn to manage their activity levels with their pain levels. Patients commonly fall into the ‘boom and bust’ cycle, in which patients push themselves intensively, which can then increase their pain levels and associated disability.^27–29^ IPS programmes will have to take into account this tendency in particular when clients are starting or returning to employment. The relationship between the client and their motivation to work was noted as important. Although few clients in this study spoke about not wanting to return to work, HCPs still viewed individuals with chronic pain as active participants in the '*sick role*'. The '*sick role'* theory describes individuals using illness as a form of social deviance, as a legitimate process to not actively participate in society, for instance through employment.^30,31^ However, this theory does not consider individual levels of disability within chronic illness, as some people may never be able to return to the norms of employment. Thinking of clients in this way could compromise the rehabilitation of individuals who do wish to gain employment. Consistent with this study's findings, previous qualitative research exploring vocational rehabilitation programmes for individuals with disabilities identified that clients had a series of concerns regarding employment, such as not being able to return to previous employment, and the unpredictability of health and its conflict with employment.^32^

The findings of this study were consistent with many of the core principles of IPS. IPS was given through one-to-one contact with ESWs, with attention paid to client preferences and needs. Although clients and ESWs acknowledged the goal of the programme was to get clients into employment, many participants spoke about the programme focusing on changing mentality, progress, and job readiness. This is an important finding as the IPS approach uses a place-and-train model, in which programmes should focus on achieving competitive employment for clients. Only one participant in this study had been on a placement, with clients mainly discussing their experiences of courses and practical advice, improving their skills for employment. Although noted that this may seem contradictory to the fundamental principle of IPS, it is acknowledged that potentially this population may need more support before embarking on a placement and paid employment.

Participants discussed another principle of IPS: the integration of medical rehabilitation. Waddell stated that HCPs have responsibility for patients returning or starting employment, as unemployment has to be sanctioned by an HCP. In the interviews, HCPs highlighted their role in helping patients stay unemployed, with the provision of doctors’ notes.^33^ HCPs and ESWs both felt more could be done for patients in this situation, connecting IPS with existing pain services. However, there was limited suggestion of how this could be achieved.

All participants identified that the current timing of the programme could pose an issue. IPS in this setting was currently offered to individuals after they had been unemployed for 2 years. However, within a population of chronic pain sufferers, prolonged unemployment leads to loss of employment opportunities, and recovery and rehabilitation back into employment is less likely.^34^ Consistent with previous literature, the participants felt that the clients would see more benefit if they could enter the programme earlier in their unemployment.^34^ Clients and ESWs also felt that the current programme's maximum time of providing support (over 12 months) was insufficient for some individuals. IPS guidance suggests that individuals should get continuous individualised support for an unlimited time.^11^ Although there are practical and financial implications of unlimited support, a potential solution would be to increase the length of the intervention programme.

### Implications for research and practice

Chronic pain significantly impacts on individuals’ quality of life and is one of the main causes of unemployment. This research highlighted that individuals with chronic pain have complex needs and a multifaceted relationship with work. The findings of this study and logic model provides a foundation of priorities and activities for future IPS interventions.^11^ IPS programmes need to identify client needs and ensure clients are engaged in returning to work. Future programmes must provide a holistic and individualised approach in supporting clients back to work. Further research is needed to tailor and evaluate the IPS for individuals with chronic pain. Integrating services, such as primary care, specialist pain services, and employment support, could lead to improved outcomes for unemployed people with chronic pain. IPS could facilitate the rehabilitation of people with chronic pain back into work. This could then lead to improved primary care provision, by moving the focus from ability to work and sick note prescribing to the management of pain.

## References

[1] Treede R-D, Rief W, Barke A (2015). A classification of chronic pain for ICD-11. Pain.

[2] Price C, Hoggart B, Olukoga O (2012). National pain audit final report 2010–2012. https://www.britishpainsociety.org/static/uploads/resources/files/members_articles_npa_2012_1.pdf.

[3] Fayaz A, Croft P, Langford RM (2016). Prevalence of chronic pain in the UK: a systematic review and meta-analysis of population studies. BMJ Open.

[4] Donaldson L (2009). 150 years of the annual report of the chief medical officer: on the state of public health 2008. https://webarchive.nationalarchives.gov.uk/20130105045448/http://www.dh.gov.uk/prod_consum_dh/groups/dh_digitalassets/documents/digitalasset/dh_096231.pdf.

[5] Waddell G, Burton AK (2004). Concepts of rehabilitation for the management of common health problems.

[6] Kposowa AJ (2001). Unemployment and suicide: a cohort analysis of social factors predicting suicide in the US national longitudinal mortality study. Psychol Med.

[7] Nylén L, Voss M, Floderus B (2001). Mortality among women and men relative to unemployment, part time work, overtime work, and extra work: a study based on data from the Swedish twin registry. Occup Environ Med.

[8] Kraut A, Mustard C, Walld R (2002). Unemployment and health care utilization. Health Effects of the New Labour Market.

[9] Söderback I, Gellman M. D, Turner J. R (2013). Functional versus vocational assessment. Encyclopedia of Behavioral Medicine.

[10] Cohen K, Bryen D, Carey A (2003). Augmentative communication employment training and supports (ACETS). Augmentative and Alternative Communication.

[11] Bond GR (1998). Principles of the individual placement and support model: empirical support. Psychiatr Rehabil J.

[12] Bond GR, Drake RE, Becker DR (2012). Generalizability of the individual placement and support (iPS) model of supported employment outside the US. World Psychiatry.

[13] Becker N, Bondegaard Thomsen A, Olsen AK (1997). Pain epidemiology and health related quality of life in chronic non-malignant pain patients referred to a Danish multidisciplinary pain center. Pain.

[14] Gatchel RJ (2004). Comorbidity of chronic pain and mental health disorders: the biopsychosocial perspective. Am Psychol.

[15] Tunks ER, Crook J, Weir R (2008). Epidemiology of chronic pain with psychological comorbidity: prevalence, risk, course, and prognosis. Can J Psychiatry.

[16] Rødevand L, Ljosaa TM, Granan LP (2017). A pilot study of the individual placement and support model for patients with chronic pain. BMC Musculoskelet Disord.

[17] Bishop FL (2015). Using mixed methods research designs in health psychology: an illustrated discussion from a pragmatist perspective. Br J Health Psychol.

[18] Guest G, Bunce A, Johnson L (2006). How many interviews are enough?. Field Methods.

[19] Denzin NK, Lincoln YS (2011). The SAGE Handbook of Qualitative Research.

[20] Mason J (2002). Qualitative Researching.

[21] Kitzinger J (1994). The methodology of focus groups: the importance of interaction between research participants. Sociol Health Illn.

[22] Bowling A (2009). Research methods in health.

[23] Wilkinson S, Joffe H, Yardley L, Marks D. F, Yardley L (2004). Qualitative data collection: interviews and focus groups. Research Methods for Clinical and Health Psychology.

[24] NVivo qualitative data analysis software [computer program] (2010). Version 10: QSR International Pty Ltd.

[25] Braun V, Clarke V (2006). Using thematic analysis in psychology. Qual Res Psychol.

[26] O'Brien BC, Harris IB, Beckman TJ (2014). Standards for reporting qualitative research: a synthesis of recommendations. Acad Med.

[27] Moseley GL (2003). A pain neuromatrix approach to patients with chronic pain. Man Ther.

[28] Ryan S (2011). Fibromyalgia: an overview and comparison of treatment options. Br J Nurs.

[29] Clark L (2009). Graded Exercise Therapy: A self-help guide for those with chronic fatigue syndrome/myalgic encephalomyelitis.

[30] Burnham JC (2014). Why sociologists abandoned the sick role concept. Hist Human Sci.

[31] Williams SJ (2005). Parsons revisited: from the sick role to...?. Health(London).

[32] van Hal L, Meershoek A, Nijhuis F (2013). Disembodied abilities: sick role and participation in 'activating' return-to-work practices. Soc Sci Med.

[33] Waddell G (1987). 1987 Volvo award in clinical sciences. A new clinical model for the treatment of low-back pain. Spine.

[34] Watson PJ, Booker CK, Moores L (2004). Returning the chronically unemployed with low back pain to employment. Eur J Pain.

